# Is cytochrome modulation the new frontier for decreasing the risk of cataract?

**DOI:** 10.4103/0253-7613.59931

**Published:** 2009-12

**Authors:** Jaykaran

**Affiliations:** Department of Pharmacology, Government Medical College, Surat - 395 001, Gujarat, India. E-mail: drjaykaran@yahoo.co.in

Sir,

I read the interesting article by Nair *et al*.,.[1] “Is cytochrome modulation the new frontier for decreasing the risk of cataract?,” wherein:

1. The authors used “Pioglitazone” as a tool for enzyme induction and “Diltiazem” for enzyme inhibition. I believe that instead of pioglitazone some other agents could have been used as the enzyme-inducing capacity of pioglitazone is questionable.

Ishida *et al*. evaluated the relation between the therapeutic effects of troglitazone and pioglitazone in steroid-induced diabetes. They measured the urinary excretion of 6[Beta]-hydroxycortisol and the ratio of 6[Beta]-hydroxycortisol to the cortisol marker of CYP3A4 induction. There was a significant increase in 6[Beta]-hydroxycortisol with troglitazone but it remained unchanged with pioglitazone.[2]

In a similar clinical study, the authors investigated the 6b-hydroxycortisol/free cortisol urinary ratio as a specific marker of CYP3A4 induction. In this study, six healthy subjects received pioglitazone 45 mg once daily on day 1 and from day 3 to day 12. The 6b-hydroxycortisol/free cortisol ratio in the urine was evaluated on day 1 and day 12. The mean (±SD) 6b-hydroxycortisol/free cortisol ratios in day 1 urine samples and day 12 urine samples were 4.99 ± 1.92 and 5.11 ± 2.01, respectively, indicating no ratio difference between day 12 versus day 1 (P = 0.29).[3] These and some other studies suggest that pioglitazone either does not induce the hepatic CYP3A4 enzyme system or is a weak inducer.[4,5]

2. The authors used ANOVA and the post hoc Tuckey for the analysis of data. Data are expressed as the percentage of total number of lenses affected. I believe that this data is nominal data and thus the “Chi-square test” could have been a better choice instead of ANOVA in this study.
